# Prescription patterns and factors influencing the use of Chinese herbal medicine among pregnant women in Taiwan: a population-based retrospective study

**DOI:** 10.1186/s12906-020-03032-0

**Published:** 2020-07-30

**Authors:** Shu-Hui Wen, Wei-Chuan Chang, Hsuan-Shu Shen, Hsien-Chang Wu

**Affiliations:** 1 Department of Public Health, College of Medicine, Tzu Chi University, Hualien, Taiwan; 2 Department of Medical Research, Buddhist Tzu Chi General Hospital, Hualien, Taiwan; 3 Department of Chinese Medicine, Buddhist Tzu Chi General Hospital, Hualien, Taiwan; 4 School of Post-baccalaureate Chinese Medicine, Tzu Chi University, Hualien, Taiwan; 5 Department of Chinese Medicine, Taipei Tzu Chi Hospital, The Buddhist Tzu Chi Medical Foundation, No. 289, Jianguo Rd., Xindian Dist, New Taipei City, 23142 Taiwan

**Keywords:** Alternative medicine, Chinese herbal medicine, Pregnant women, Peripartum, Perinatal, Traditional Chinese medicine

## Abstract

**Background:**

The use of Chinese herbal medicine (CHM) has been widely promoted as a natural and safe way to treat illness during pregnancy. However, prescription patterns and factors influencing its use are largely unknown. Therefore, we conducted a population-based study to address these questions.

**Methods:**

Pregnant women aged 18–50 years were selected from Taiwan’s National Health Insurance Research Database between 2001 to 2011. CHM prescriptions and diagnostic records were collected. Demographic data and pre-existing diseases were compared between CHM users and non-users. A multivariate logistic regression analysis was performed to identify possible factors influencing the use of CHM during pregnancy.

**Results:**

A total of 81,873 eligible prescription records were identified, and 16,553 pregnant women were prescribed CHM during pregnancy, yielding a CHM prescription rate of 20.2%. The three most frequently used herbs were Scutellariae Radix (Huang Qin) (4.4%), Eucommiae cortex (Du Zhong) (2.5%), and Atractylodes Rhizome (Bai Zhu) (2.4%). The most frequently used herbal formulae were Dang-Guei-Shao-Yao-San (4.1%), Jia-Wei-Xiao-Yao-San (3.5%), and Xiang-Sha-Liu-Jun-Zi-Tang (2.6%). Multivariate logistic regression revealed that subjects with an older age, a university education, a pre-pregnancy history of CHM use, asthma, chronic renal disease, and cardiac valvular disease and living in a residential area other than northern Taiwan had an increase in adjusted odds ratio for CHM use during pregnancy.

**Conclusions:**

In this population-based study, we found that demographic factors and pre-existing diseases were associated with the use of CHM among pregnant women. It is worth noting that Leonuri Herba (Yi Mu Cao) and Shao-Fu-Zhu-Yu-Tang should be used with caution in the first trimester. Further research is needed to explore the safety and effectiveness of the use of CHM in pregnant women.

## Background

Chinese herbal medicine (CHM) has been widely used as a natural and safe way to treat illness during pregnancy [1]. The reported prevalence of medicinal herb usage during pregnancy ranges from 12 to 58% in various countries [2–6]. In Taiwan, 34% of Taiwanese women consume at least one type of CHM during pregnancy [5]. In clinical settings, women tend to take CHM as an alternative treatment for a variety of pregnancy diseases, such as vomiting during pregnancy, hypertension, and miscarriage [7, 8]. However, the prescription patterns and factors influencing the use of CHM during pregnancy are largely unknown.

Previous studies have shown that there are differences in the socio-demographic and health-related characteristics between users of complementary and alternative medicine (CAM) and non-users [9]. For example, subjects with old age, females, high socioeconomic groups, and those with a high education level and health problems are prone to use CAM in countries other than Taiwan [9–11]. Similar findings have also been reported by investigators who used the National Health Insurance Research Database (NHIRD) in Taiwan [12, 13]. However, factors influencing the use of CHM in the subset of pregnant women remain unclear.

Taiwan’s health authority maintains traditional Chinese medicine (TCM) under the auspices of the National Health Insurance (NHI) Service and enhances CHM quality control management to ensure the health and medical care for the public. NHIRD was established by the NHI as a universal health insurance program since 1995 in Taiwan. The database covers more than 99.6% of the Taiwanese population [14, 15]. Particularly, pregnant women who need CHM treatment are required to obtain prescriptions from TCM physicians. Thus, NHIRD is a large database that comprehensively includes demographic and clinical data of all pregnant women who receive TCM. An understanding of the prescription patterns may be helpful for monitoring and evaluating the adequacy of CHM usage among pregnant women. In addition, an analysis of the factors related to CHM use may provide essential information to clinicians regarding the profiles of pregnant women who are more likely to use CHM. In this study, we aimed to investigate the prescription patterns and factors influencing the use of CHM during pregnancy by using this dataset. To achieve this goal, the prescription patterns in relation to diagnoses were evaluated. Factors influencing the use of CHM were identified by comparisons of variables between the CHM users and non-users.

## Methods

### Data resources

We conducted a retrospective population-based study using the longitudinal health insurance database (LHID) for the period from 2001 to 2011, which was provided by the Health and Welfare Statistics Application Centre of the Ministry of Health and Welfare in Taiwan. The LHID comprises medical claims data of more than 2 million beneficiaries who have been randomly sampled since the year 2000 from registries of all NHI enrolees. The LHID contains data on patient demographic characteristics, outpatient and inpatient visit records, drug prescriptions (including CHM), and International Classification of Disease-Clinical Modification (9th revision) (ICD-9-CM) diagnoses. The information on diagnosis codes of the database have been validated by a previous study [16]. This study was approved by the Institutional Review Board of Taipei Tzu Chi Hospital in Taipei, Taiwan (Protocol No. 05-W03–050).

### Study sample and measured variables

**Fig. 1:**
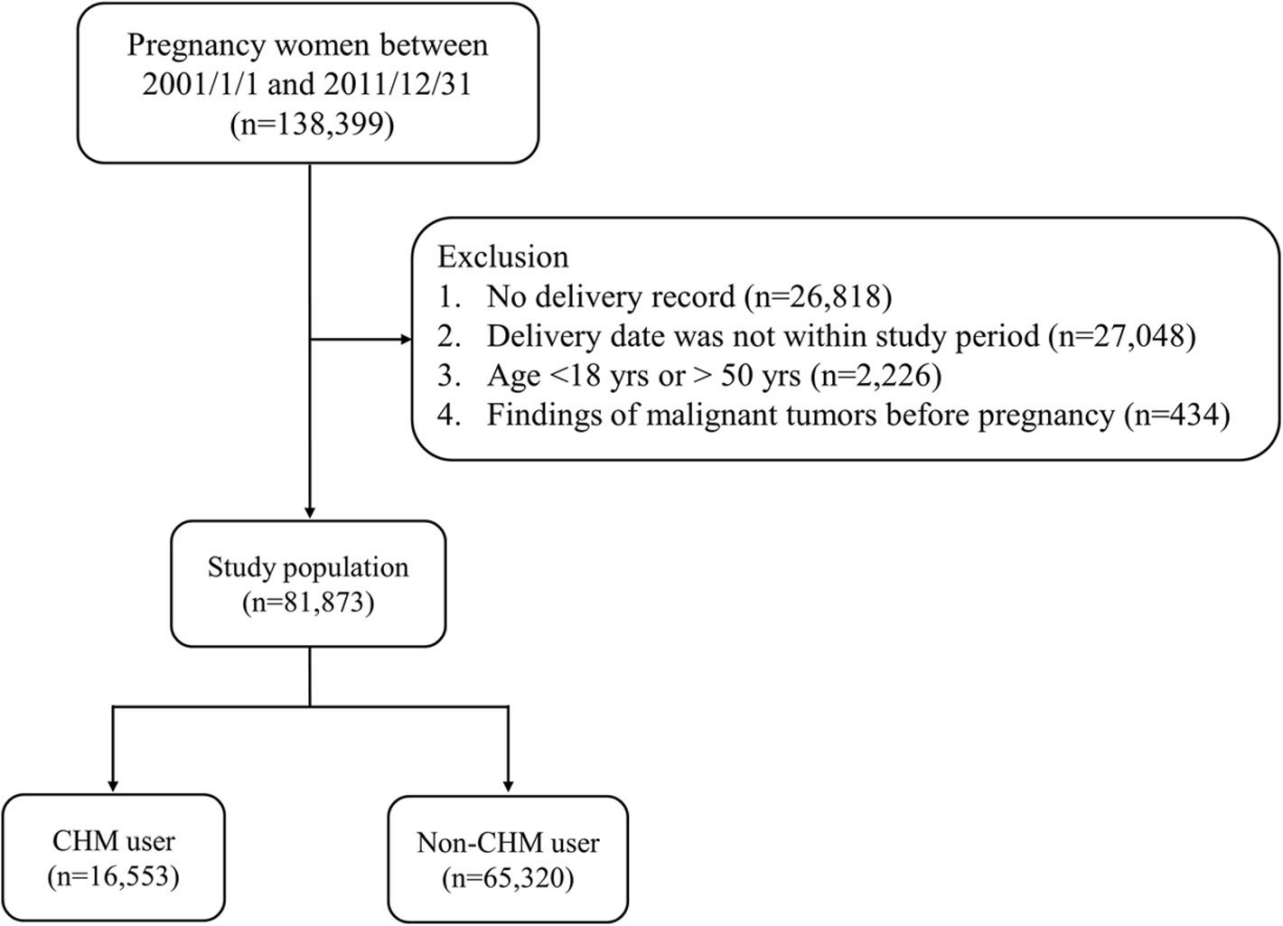
Flow chart of the study population

Medical records of pregnant women were selected from the LHID for the period between 2001 and 2011 using the following inclusion criteria: (1) aged between 18 and 50 years; (2) confirmed as pregnant (ICD-9-CM codes: V22 and V23) via outpatient or inpatient visit; and (3) a birth outcome of term birth (ICD-9-CM codes: V27.0, V27.2, V27.3, V27.5, V27.6, V30-V37, V39, 650), preterm birth (ICD-9-CM code: 765.1), abortion (ICD-9-CM codes: 632; ICD_OP_CODE: 690, 695, 750), or stillbirth (ICD-9-CM codes: V27.1, V27.4, V27.7, 656.4). We estimated the start date of pregnancy according to the initial prenatal visit which represents its specific timing during pregnancy. The duration of pregnancy was defined as the time between the start date of pregnancy and the date of the end of pregnancy due to a live birth, abortion, or stillbirth. We excluded cases based on the following criteria: male gender, no record of birth outcome, age outside of the specified inclusion range, a pre-pregnancy malignant tumour history, or incomplete prenatal visits. A total of 81,873 eligible pregnant women were identified. Figure 1 shows the flowchart of the study.

Among these, 16,553 pregnant women (20.2%) used CHM which was determined by correlating TCM outpatient visits and CHM prescription records. All CHM prescription records were classified as either single herbs or herbal formulae. Prescription patterns and primary diagnostic codes associated with TCM outpatient visits were presented in terms of three (first, second, third) trimester groups. To understand the prescription patterns and safety of CHM use during human embryogenesis (6–12 weeks of gestation), we analysed all gynaecological diagnoses and prescriptions recorded both before and after the first prenatal visit. That is, we assessed common diagnostic codes and CHM use before or after the first prenatal visit only among women with obstetrics-related TCM records (ICD-9-CM code: 610–677 or A code: A370-A394). Women may have consulted with their TCM physicians prior to a confirmed pregnancy, before their first prenatal visit. Thus, it was important to assess CHM use during this period.

Sample characteristics, including age at pregnancy, education level (below junior, senior, university, or above), income (monthly income in new Taiwan dollars [NTD]), residential area (northern, middle, southern, or eastern Taiwan), prior usage (whether she had used CHM within the 6 months before pregnancy), and maternal comorbidity, were also collected. Maternal comorbidities were diagnosed with ICD-9-CM codes (Supplementary Table 2) from at least one ambulatory visit or inpatient claim within the year prior to pregnancy [17].

### Statistical analyses

Descriptive statistics (numbers and percentages) were provided for the distribution of primary diagnostic codes and CHM use. Outpatient TCM records from pregnancy were further restricted to obstetrics-related codes. The 10 most common primary diagnostic codes and CHMs were presented for the three trimesters, as well as before or after the first prenatal visit. A logistic regression analysis was performed to examine the factors associated with CHM use during pregnancy. The covariates were age at pregnancy, education level, income, residential area, prior usage, and maternal comorbidity. Odds ratios (ORs) and accompanying 95% confidence intervals (CIs) were provided. Data analyses were performed with SAS version 9.3 (SAS Institute, Inc., Cary, NC, USA).

## Results

### Sample characteristics

A total of 16,553 pregnant women received CHM during pregnancy (termed ‘CHM users’ hereafter), yielding a CHM utilization rate of 20.2%. The average duration between the estimated start date of pregnancy and the first CHM prescription record was 62.8 days. In total, 81.7% of the women received their first CHM prescription during the first trimester. The average duration of CHM use was 19.8 days during pregnancy.

Some characteristics differed significantly between CHM users and non-users (Table 1). CHM users were slightly older, had longer pregnancies, and had higher education and income levels (*P* < 0.05). More CHM users lived in middle and southern Taiwan than non-users. Slightly higher percentages of maternal comorbidities such as asthma, chronic renal disease, Sickle cell disease, and cardiac valvular disease were present in CHM users compared to non-users (*P* < 0.05). Finally, CHM use 6 months prior to pregnancy was more common among TCM users than non-users (63.1% vs. 22.7%, *P* < 0.05).

**Table 1:** Characteristics between pregnant women with and without CHM use (*n* = 81,873)

Characteristics	CHM user *n = 16,553*	Non-CHM user *n = 65,320*	*p*-value
Age (years)	28.6 ± 4.6	28.0 ± 4.8	< 0.001
Duration of pregnancy (days)	266.3 ± 63.5	255.9 ± 69.8	< 0.001
Education			< 0.001
Below Junior	3582 (22.1)	16,203 (25.5)	
Senior	7148 (44.1)	28,521 (44.8)	
University or above	5489 (33.8)	18,912 (29.7)	
Income level (1000 NTD per unit)	24.7 ± 16.2	23.9 ± 16.9	< 0.001
< 20,000	5858 (41.7)	24,189 (44.5)	< 0.001
20,000-39,999	5685 (40.4)	21,031 (38.7)	
> 40,000	2512 (17.9)	9147 (16.8)	
Residential area			< 0.001
Northern	5565 (33.7)	28,861 (44.3)	
Middle	4621 (27.9)	11,890 (18.3)	
Southern	5538 (33.6)	20,467 (31.4)	
Eastern	784 (4.8)	3916 (6.0)	
Maternal Comorbidity			
Asthma	352 (2.1)	1036 (1.6)	< 0.001
Chronic ischemic heart disease	50 (0.3)	149 (0.2)	0.084
Chronic renal disease	119 (0.7)	256 (0.4)	< 0.001
Congenital heart disease	13 (0.1)	52 (0.1)	0.965
Preexisting diabetes mellitus	118 (0.7)	402 (0.6)	0.159
Preexisting hypertension	77 (0.5)	303 (0.5)	0.982
Sickle cell disease	37 (0.2)	88 (0.1)	0.009
Cardiac valvular disease	189 (1.1)	441 (0.7)	< 0.001
Placenta previa	6 (0.1)	36 (0.1)	0.338
Gestational hypertension	12 (0.1)	33 (0.1)	0.281
CHM use at 6 months before first pregnancy diagnosis	10,449 (63.1)	14,812 (22.7)	< 0.001

Data are shown as n (%), or mean ± standard deviation

*CHM* Chinese herbal medicine, *NTD* New Taiwan dollar

### Prescription patterns of CHM users

The top 10 single herbs, herbal formulae, and primary diagnostic codes identified among CHM users are listed in Supplementary Table 1. For all CHM records, the top three most common primary diagnostic codes were abnormal bleeding from the female genital tract (12%), acute nasopharyngitis (11.2%), and cough (5.2%). The most commonly used single herbs were Scutellariae Radix (Huang Qin) (4.4%), Eucommiae cortex (Du Zhong) (2.5%), Atractylodes Rhizome (Bai Zhu) (2.4%), Cyperi Rhizoma (Xiang Fu) (2.1%), Cuscutae Semen (Tu Si Zi) (2%), and Dipsaci Radix (Xu Duan) (2%). The most commonly used herbal formulae were Dang-Guei-Shao-Yao-San (4.1%), Jia-Wei-Xiao-Yao-San (3.5%), Xiang-Sha-Liu-Jun-Zi-Tang (2.6%), Bao-Chan-Wu-You-Fang (2.6%), Yin-Qiao-San (2.2%), and Xin-Yi-Qing-Fei-Tang (2%), which together accounted for 17% of the herbal formulae prescribed.

There were also differences identified among the three trimester groups regarding diagnostic codes and prescription patterns (Table 2). For instance, the most common diagnostic code issued in the first trimester was abnormal bleeding from the female genital tract (17.2%), while acute nasopharyngitis was most common during the second (11.9%) and third (16.5%) trimesters. Hypertension complicating pregnancy, childbirth, and the puerperium (6.9%) and sleep disturbances (3.1%) became more common in the third trimester. Increased use of Atractylodes Rhizome (Bai Zhu) and Platycodi Radix (Jie Geng) was observed in the second and third trimesters. In particular, single herbs such as Leonuri Herba (Yi Mu Cao) and Corydalis Rhizoma (Yan Hu Su) and herbal formulae such as Wen-Jing-Tang and Ban-Xia-Xie-Xin-Tang were used only during the first trimester.

**Table 2:** Top 10 single herbal, Herbal formulae and diagnosis and prescription in CHM users at three trimesters

First trimester		Second trimester		Third trimester	
	Frequency (%)		Frequency (%)		Frequency (%)
**Diagnosis**					
Abnormal bleeding from female genital tract	34,960 (17.2)	Acute nasopharyngitis	9311 (11.9)	Acute nasopharyngitis	6801 (16.5)
Acute nasopharyngitis	18,080 (8.9)	Abnormal bleeding from female genital tract	8467 (10.8)	Cough	3618 (8.8)
Disorders of function of stomach	9446 (4.7)	Cough	4940 (6.3)	Hypertension complicating pregnancy, childbirth, and the puerperium	2846 (6.9)
Excessive vomiting in pregnancy	7445 (3.7)	Headache	3102 (4.0)	Disorders of function of stomach	1826 (4.4)
Headache	7370 (3.6)	Hypertension complicating pregnancy, childbirth, and the puerperium	3023 (3.9)	Sleep disturbances	1279 (3.1)
Constipation	7339 (3.6)	Sleep disturbances	2909 (3.7)	Headache	1228 (3.0)
Cough	7177 (3.5)	Disorders of function of stomach	2885 (3.7)	Low back pain	1191 (2.9)
Sleep disturbances	6836 (3.4)	Constipation	2594 (3.3)	Constipation	1191 (2.9)
Female infertility associated with anovulation	6715 (3.3)	Allergic rhinitis	2337 (3.0)	Pruritus and related conditions	1188 (2.9)
Dysmenorrhea	5432 (2.7)	Excessive vomiting in pregnancy	2214 (2.8)	Chronic pharyngitis and nasopharyngitis	1137 (2.7)
**Single herbal**					
*Scutellariae Radix* (Huang Qin)	4023 (3.5)	*Scutellariae Radix* (Huang Qin)	2096 (4.7)	*Scutellariae Radix* (Huang Qin)	1504 (6.3)
*Cyperi Rhizoma* (Xiang Fu)	3191 (2.7)	*Eucommiae cortex* (Du Zhong)	1288 (2.9)	*Atractylodes Rhizome* (Bai Zhu)	845 (3.5)
*Eucommiae cortex* (Du Zhong)	2894 (2.5)	*Atractylodes Rhizome* (Bai Zhu)	1133 (2.5)	*Eucommiae cortex* (Du Zhong)	679 (2.8)
*Cuscutae Semen* (Tu Si Zi)	2740 (2.4)	*Dipsaci Radix* (Xu Duan)	961 (2.1)	*Platycodi Radix* (Jie Geng)	610 (2.5)
*Corydalis Rhizoma* (Yan Hu Su)	2426 (2.1)	*Platycodi Radix* (Jie Geng)	864 (1.9)	*Fritillariae Thunbergii Bulbus* (Bei Mu)	549 (2.3)
*Leonuri Herba* (Yi Mu Cao)	2353 (2.0)	*Cyperi Rhizoma* (Xiang Fu)	843 (1.9)	*Dipsaci Radix* (Xu Duan)	536 (2.2)
*Dipsaci Radix* (Xu Duan)	2280 (1.9)	*Cuscutae Semen* (Tu Si Zi)	826 (1.8)	*Glycyrrhizae Radix et Rhizoma* (Gan Cao)	446 (1.9)
*Atractylodes Rhizome* (Bai Zhu)	2154 (1.8)	*Fritillariae Thunbergii Bulbus* (Bei Mu)	690 (1.5)	*Ophiopogonis Radix* (Mai Men Dong)	430 (1.8)
*Rhei Radix et Rhizoma* (Da Huang)	1840 (1.6)	*Glycyrrhizae Radix et Rhizoma* (Gan Cao)	683 (1.5)	*Taxilli Herba* (Sang Ji Sheng)	407 (1.7)
*Platycodi Radix* (Jie Geng)	1670 (1.4)	*Ophiopogonis Radix* (Mai Men Dong)	680 (1.5)	*Scrophulariae Radix* (Xuan Shen)	371 (1.6)
**Herbal formulae**					
Dang-Gui-Shao-Yao-San	3805 (4.4)	Dang-Gui-Shao-Yao-San	1447 (4.3)	Bao-Chan-Wu-You-Fang	898 (5.2)
Jia-Wei-Xiao-Yao-San	3781 (4.3)	Jia-Wei-Xiao-Yao-San	1047 (3.1)	Dang-Gui-Shao-Yao-San	648 (3.7)
Xiang-Sha-Liu-Jun-Zi-Tang	2240 (2.6)	Bao-Chan-Wu-You-Fang	823 (2.5)	Yin-Qiao-San	599 (3.4)
Wen-Jing-Tang	1836 (2.1)	Yin-Qiao-San	749 (2.2)	Xin-Yi-Qing-Fei-Tang	517 (3.0)
Ban-Xia-Xie-Xin-Tang	1601 (1.8)	Xin-Yi-Qing-Fei-Tang	711 (2.1)	Ma-Xing-Gan-Shi-Tang	403 (2.3)
Ping-Wei-San	1530 (1.8)	Xiang-Sha-Liu-Jun-Zi-Tang	677 (2.0)	Xiang-Sha-Liu-Jun-Zi-Tang	370 (2.1)
Yin-Qiao-San	1529 (1.8)	Chuan-Qiong-Cha-Tiao-San	622 (1.9)	Gan-Lu-Yin	366 (2.1)
Gui-Zhi-Fu-Ling-Wan	1521 (1.7)	Gui-Pi-Tang	557 (1.7)	Sang-Ju-Yin	342 (2.0)
Chuan-Qiong-Cha-Tiao-San	1473 (1.7)	Ma-Zi-Ren-Wan	541 (1.6)	Xiao-Chai-Hu-Tang	335 (1.9)
Ma-Zi-Ren-Wan	1430 (1.6)	Ping-Wei-San	534 (1.6)	Jia-Wei-Xiao-Yao-San	327 (1.9)

We further limited our assessments to obstetrics-related records and CHM usage either before or after the first prenatal visit (Table 3). We found that abnormal bleeding from the female genital tract (53%), infertility (10.4%), and dysmenorrhea (8.3%) were the three most common primary diagnoses encountered before the first prenatal visit. After the first prenatal visit, abnormal bleeding from the female genital tract (53.3%), dysmenorrhea (9.0%), and hypertension complicating pregnancy, childbirth, and the puerperium (7.7%) were the three most common primary diagnoses. Before the first prenatal visit, the most frequently used single herb prescribed was Cyperi Rhizoma (Xiang Fu) (5%), followed by Cuscutae Semen (Tu Si Zi) (4.5%) and Leonuri Herba (Yi Mu Cao) (4.2%). After the first prenatal visit, the top three single herbs prescribed differed from those prescribed before the first prenatal visit. Cyperi Rhizoma (Xiang Fu) (4.7%) was the most frequently prescribed single herb, followed by Leonuri Herba (Yi Mu Cao) (4.1%) and Corydalis Rhizoma (Yan Hu Su) (3.2%). The top three most frequently prescribed herbal formulae were Dang-Guei-Shao-Yao-San, Jia-Wei-Xiao-Yao-San, and Wen-Jing-Tang, irrespective of the first prenatal visit.

**Table 3:** Top 10 obstetrics related diagnosis and prescription in pregnancy women before and after the first prenatal visit

	Before prenatal Visit		After prenatal Visit	
Diagnosis n (%)^a^	Abnormal bleeding from female genital tract	28,738 (53.0)	Abnormal bleeding from female genital tract	55,460 (53.3)
	Infertility	5649 (10.4)	Dysmenorrhea	9403 (9.0)
	Dysmenorrhea	4494 (8.3)	Hypertension complicating pregnancy, Childbirth, and the puerperium	8014 (7.7)
	Excessive vomiting in pregnancy	4087 (7.5)	Excessive vomiting in pregnancy	6729 (6.5)
	Hypertension complicating pregnancy, Childbirth, and the puerperium	2843 (5.2)	Infertility	6364 (6.1)
	Leukorrhea	2131 (3.9)	Leukorrhea	5311 (5.1)
	Hemorrhage in early pregnancy	1213 (2.2)	Other complications of pregnancy	2350 (2.3)
	Endometriosis	905 (1.7)	Hemorrhage in early pregnancy	1525 (1.5)
	Premenstrual tension syndromes	885 (1.6)	Premenstrual tension syndromes	1284 (1.2)
	Other complications of pregnancy	622 (1.1)	Endometriosis	963 (0.9)
Single herbal n (%)^b^	*Cyperi Rhizoma* (Xiang Fu)	1724 (5.0)	*Cyperi Rhizoma* (Xiang Fu)	3109 (4.7)
	*Cuscutae Semen* (Tu Si Zi)	1555 (4.5)	*Leonuri Herba* (Yi Mu Cao)	2686 (4.1)
	*Leonuri Herba* (Yi Mu Cao)	1450 (4.2)	*Corydalis Rhizoma* (Yan Hu Su)	2104 (3.2)
	*Eucommiae cortex* (Du Zhong)	1161 (3.4)	*Eucommiae cortex* (Du Zhong)	2027 (3.1)
	*Scutellariae Radix* (Huang Qin)	1090 (3.2)	*Cuscutae Semen* (Tu Si Zi)	1986 (3.0)
	*Corydalis Rhizoma* (Yan Hu Su)	943 (2.7)	*Scutellariae Radix* (Huang Qin)	1946 (3.0)
	*Dipsaci Radix* (Xu Duan)	927 (2.7)	*Salviae Miltiorrhizae Radix ET Rhizoma* (Dan Shen)	1479 (2.3)
	*Salviae Miltiorrhizae Radix ET Rhizoma* (Dan Shen)	720 (2.1)	*Dipsaci Radix* (Xu Duan)	1439 (2.2)
	*Atractylodes Rhizome* (Bai Zhu)	681 (2.0)	*Atractylodes Rhizome* (Bai Zhu)	1207 (1.8)
	*Ligustri Lucidi Fructus* (Nu Zhen Zi)	598 (1.7)	*Rhei Radix et Rhizoma* (Da Huang)	924 (1.4)
Herbal formulae n (%)^c^	Dang-Gui-Shao-Yao-San	2082 (8.9)	Jia-Wei-Xiao-Yao-San	3599 (7.9)
	Jia-Wei-Xiao-Yao-San	1844 (7.9)	Dang-Gui-Shao-Yao-San	3492 (7.7)
	Wen-Jing-Tang	1294 (5.5)	Wen-Jing-Tang	2213 (4.9)
	Gui-Zhi-Fu-Ling-Wan	896 (3.8)	Gui-Zhi-Fu-Ling-Wan	1953 (4.3)
	Shao-Fu-Zhu-Yu-Tang	602 (2.6)	Gui-Pi-Tang	1292 (2.8)
	Gui-Pi-Tang	581 (2.5)	Shao-Fu-Zhu-Yu-Tang	1286 (2.8)
	Xiang-Sha-Liu-Jun-Zi-Tang	580 (2.5)	Qiong-Gui-Jiao-Ai-Tang	978 (2.1)
	Si-Wu-Tang	495 (2.1)	Si-Wu-Tang	879 (1.9)
	Qiong-Gui-Jiao-Ai-Tang	483 (2.1)	Bao-Chan-Wu-You-Fang	847 (1.9)
	Zuo-Gui-Wan	481 (2.1)	Long-Dan-Xie-Gan-Tang	833 (1.8)

^a^the total numbers of diagnosis before and after prenatal visit were 57,941 and 111,403, respectively

^b^the total number of prescriptions for single herbal before and after prenatal visit were34,454 and 65,769, respectively

^c^the total number of prescriptions for herbal formulae before and after prenatal visit were 23,487 and 45,634, respectively

### Factors influencing the use of CHM during pregnancy

A multivariate logistic regression model was used to examine the factors associated with CHM use during pregnancy (Table 4). The following factors were associated with a greater likelihood of using CHM: older pregnant women (OR = 1.03, 95% CI: 1.02–1.03), women with a university education (OR = 1.16, 95% CI: 1.09–1.23), those who did not live in northern Taiwan (middle Taiwan dwellers: OR = 1.78, 95% CI: 1.69–1.88; southern Taiwan dwellers: OR = 1.36, 95% CI: 1.30–1.43; eastern Taiwan dwellers OR = 1.14, 95% CI: 1.04–1.26), and those who had pre-pregnancy experience with CHM (OR = 5.69, 95% CI: 5.46–5.93). Women with asthma (OR = 1.23, 95% CI: 1.05–1.43), chronic renal disease (OR = 1.33, 95% CI: 1.02–1.73), or cardiac valvular disease (OR = 1.54, 95% CI: 1.25–1.91) were also more likely to use CHM during pregnancy.

**Table 4:** Associating factors with the use of CHM during pregnancy (*n* = 81,873)

Characteristics	aOR (95% CI)	*P*-value
Age	1.03 (1.02–1.03)	< 0.001
Education		
Below Junior	ref	
Senior	1.04 (0.98–1.09)	0.080
University or above	1.16 (1.09–1.23)	< 0.001
Residential area		
Northern	ref	
Middle	1.78 (1.69–1.88)	< 0.001
Southern	1.36 (1.30–1.43)	0.004
Eastern	1.14 (1.04–1.26)	0.001
CHM use before 6 months of pregnancy	5.69 (5.46–5.93)	< 0.001
Asthma	1.23 (1.05–1.43)	0.009
Chronic renal disease	1.33 (1.02–1.73)	0.034
Cardiac valvular disease	1.54 (1.25–1.91)	< 0.001

Multiple logistic regression analysis was used to calculate adjusted odds ratio (aOR). Adjusted factors included age, education, income, residential area, CHM use before 6 months of pregnancy, asthma, chronic ischemic heart disease, chronic renal disease, congenital heart disease, preexisting diabetes mellitus, preexisting hypertension, sickle cell disease, cardiac valvular disease, placenta previa, and gestational hypertension

*CHM* Chinese herbal medicine

## Discussion

Our research describes the CHM prescription patterns in a population of pregnant women in Taiwan and reveals discrepancies between diagnoses and CHM usage during each of the three trimesters. We found that the CHM utilization rate during pregnancy was 20.2%. This rate is similar to those reported in prior prospective cohort studies [5, 18] and a study in Taiwan [7], but lower than those reported previously in Norway (39.7%) [19] and mainland China (43.5%) [1]. This may be because the NHIRD only contains medical records for cases diagnosed and drugs prescribed by TCM physicians. Thus, herbal medicines purchased and consumed by patients on their own were not included. As for factors related to CHM use, pregnant women with a higher level of education and larger income had greater rates of CHM usage. This may be because CHM users usually have a higher socioeconomic status and marry later in life, thus requiring menstruation regulation to increase the chance of pregnancy [5]. Additionally, we found that women treated with CHM or those who had chronic diseases before pregnancy preferred the use of CHM to treat their illness; this is similar to findings reported in a previous study of Chinese CHM users [20].

Among pregnant women treated with CHM, the most common diagnoses were related to respiratory system diseases (such as acute nasopharyngitis, cough, allergic rhinitis, etc.). This is consistent with the findings of previous research [7]. Other common diagnoses included gastrointestinal diseases, pregnancy-related vomiting or bleeding, and pregnancy complicated by hypertension. Recent studies have demonstrated that TCM is also therapeutically effective not just for upper respiratory tract infections and gastrointestinal diseases, but also for bleeding during pregnancy, pregnancy-related vomiting, and pregnancy-induced hypertension [8, 21–24].

A previous study [7] reported data of diagnoses during the three trimesters, but the data were based on the systems of diseases (i.e., respiratory or circulatory system). We also reported data regarding diagnoses and prescriptions during the three trimesters, but our data were based on more accurate diagnoses of single diseases. The data presented herein indicate that TCM is increasingly being used to treat pregnancy-related complications and symptoms. The diagnoses of these common diseases are consistent with the changes in physiology and pathology that occur during pregnancy [25] and reveal which diseases may most correlate with TCM treatment during the three trimesters. Due to recent trends toward further refinement of modern medicine, obstetricians often focus solely on obstetrical disease, casting aside the other common medical conditions faced by pregnant women. TCM treatment may have clinically curative effects for symptoms or diseases presenting in pregnancy, reduce the side effects and frequency of conventional medicine use, and achieve improved health in pregnant women. A review of relevant literature shows that, when compared with conventional medicine alone, combined use of TCM and standard treatments exhibits superior results and fewer side effects [8]. Furthermore, this research reveals the proportion of diseases diagnosed among pregnant women in Taiwan and may thus be used to guide clinical practices in the country.

Certain herbal formulae were identified as the most commonly used by pregnant women in the present study. Jia-Wei-Xiao-Yao-San can be used to treat anxiety, irritability, stress, depression, premenstrual tension, climacteric syndrome, and infertility [26–28]. In particular, the anxiolytic effects of this herbal formula are thought to act through neurosteroid synthesis [29, 30]. Xiang-Sha-Liu-Jun-Zi-Tang has long been used in clinical practice to treat gastrointestinal discomfort such as nausea and vomiting, emaciation, and anorexia. A meta-analysis found that Xiang-Sha-Liu-Jun-Zi-Tang could improve symptoms significantly more than prokinetic drugs in the treatment of functional dyspepsia [31]. Atractylodes Rhizome (Bai Zhu) works together with Scutellariae Radix (Huang Qin) to prevent miscarriage through inhibition of maternal-fetal interface immunity [32]. Past research has shown that Cortex Eucommiae (Du Zhong) contains isoflavonoids, which were reported to exhibit phytoestrogenic and androgenic properties, likely related to the optimization of sex hormone activity in the maternal body [33].

Past studies have demonstrated that physicians and pregnant women should pay particular attention to the safety and adverse effects of drugs and herbs during pregnancy [34, 35]. We found that 81.7% of patients received their first CHM prescription during the first trimester. During the first prenatal visit period, abnormal bleeding from the female genital tract (53%) and dysmenorrhea were the most common diagnoses.

Research has confirmed that some habitual abortion cases are associated with increased blood viscosity [36]. Moreover, habitual abortions may also be associated with autoimmune antibody abnormalities, especially in anti-cardiolipin antibodies (ACLs). The pathological mechanism underlying the contribution of ACLs to habitual abortion is mainly related to thrombosis in the placental blood vessels and uterine spiral arterials, fibroid necrosis, and atherosclerosis [37]. Aspirin or heparin are thus often used to inhibit platelet aggregation and anticoagulation [38].

Our study revealed that most prescriptions and medications met the pharmacopeia’s use specifications, and TCM physicians used them according to their appropriate diagnostic prescriptions. However, according to the warning from the Herbal Pharmacopeia [39], leonuri Herba (Yi Mu Cao) and Shao-Fu-Zhu-Yu-Tang should be used with caution during pregnancy due to possible adverse effects such as increased contractile frequency and activity of the uterus [40]. We speculate that these herbs are likely to be used by older, highly educated women to treat infertility caused by dysmenorrhea and endometriosis disease [41, 42]. Therefore, we recommend that TCM physicians should be cautious when prescribing CHM for pregnant women. Practitioners should avoid excess dosing and long-term use in these patients. The use and prescription patterns of CHM in pregnancy warrant further study and analysis.

Despite our findings, the present study also had some limitations. First, herbs purchased and used by patients themselves were not contained in the NHI records and thus were not included. Therefore, the frequency of TCM utilisation may have been underestimated. Second, no laboratory data or imaging findings of labour outcomes were available in this database; thus, treatment efficacy could not be determined. To establish the underlying biological mechanisms of action of these CHMs, further research should be conducted.

## Conclusions

In this population-based study, we found that demographic factors and pre-existing diseases were associated with the use of CHM in pregnant women. It is worth noting that Leonuri Herba (Yi Mu Cao) and Shao-Fu-Zhu-Yu-Tang should be used with caution in the first trimester. Further investigations should be directed to the safety and effectiveness of the use of CHM in pregnant women.

## Supplementary information

**Additional file 1: Table S1.** Top 10 diagnosis and prescription in CHM users during pregnancy during follow up. **Table S2.** The ICD-9-CM codes for maternal comorbidities.

## Data Availability

The data that support the findings of this study are available from the Bureau of National Health Insurance, Department of Health, and managed by the Health and Welfare Statistics Application Centre, Ministry of Health and Welfare but restrictions apply to the availability of these data, which were used under license for the current study, and so are not publicly available.

## Abbreviations

CAM: Complementary and alternative medicine
CHM: Chinese herbal medicine
TCM: Traditional Chinese medicine
NHI: National Health Insurance
ICD-9-CM: International Classification of Disease-Clinical Modification (9th revision)
LHID: Longitudinal health insurance database
ORs: Odds ratios
CIs: Confidence intervals
ACLs: Anti-cardiolipin antibodies

## References

[1] Tang L, Lee AH, Binns CW, Hui YV, Yau KKW (2016). Consumption of Chinese herbal medicines during pregnancy and postpartum: a prospective cohort study in China. Midwifery.

[2] Kennedy DA, Lupattelli A, Koren G, Nordeng H (2013). Herbal medicine use in pregnancy: results of a multinational study. BMC Complement Altern Med.

[3] Frawley J, Adams J, Steel A, Broom A, Gallois C, Sibbritt D (2015). Women’s use and self-prescription of herbal medicine during pregnancy: an examination of 1,835 pregnant women. Womens Health Issues.

[4] Ahmed M, Hwang JH, Choi S, Han D (2017). Safety classification of herbal medicines used among pregnant women in Asian countries: a systematic review. BMC Complement Altern Med.

[5] Chuang CH, Chang PJ, Hsieh WS, Tsai YJ, Lin SJ, Chen PC (2009). Chinese herbal medicine use in Taiwan during pregnancy and the postpartum period: a population-based cohort study. Int J Nurs Stud.

[6] Ong CO, Chan LY, Yung PB, Leung TN (2005). Use of traditional Chinese herbal medicine during pregnancy: a prospective survey. Acta Obstet Gynecol Scand.

[7] Yeh HY, Chen YC, Chen FP, Chou LF, Chen TJ, Hwang SJ (2009). Use of traditional Chinese medicine among pregnant women in Taiwan. Int J Gynaecol Obstet.

[8] Li L, Leung PC, Chung TK, Wang CC (2014). Systematic review of Chinese medicine for miscarriage during early pregnancy. Evid Based Complement Alternat Med.

[9] Wiles J, Rosenberg MW (2001). ‘Gentle caring experience’. Seeking alternative health care in Canada. Health Place.

[10] Astin JA (1998). Why patients use alternative medicine: results of a national study. JAMA.

[11] Kemppainen LM, Kemppainen TT, Reippainen JA, Salmenniemi ST, Vuolanto PH (2018). Use of complementary and alternative medicine in Europe: health-related and sociodemographic determinants. Scand J Public Health.

[12] Chen FP, Chen TJ, Kung YY, Chen YC, Chou LF, Chen FJ, Hwang SJ (2007). Use frequency of traditional Chinese medicine in Taiwan. BMC Health Serv Res.

[13] Hsieh SC, Lai JN, Lee CF, Hu FC, Tseng WL, Wang JD (2008). The prescribing of Chinese herbal products in Taiwan: a cross-sectional analysis of the national health insurance reimbursement database. Pharmacoepidemio Drug Saf.

[14] Cheng SH, Chiang TL (1997). The effect of universal health insurance on health care utilization in Taiwan. Results from a natural experiment. JAMA.

[15] Lin LY, Warren-Gash C, Smeeth L, Chen PC (2018). Data resource profile: the National Health Insurance Research Database (NHIRD). Epidemiol Health.

[16] Hsieh CY, Su CC, Shao SC, Sung SF, Lin SJ, Kao Yang YH, Lai EC (2019). Taiwan’s National Health Insurance Research Database: past and future. Clin Epidemiol.

[17] Bateman BT, Mhyre JM, Hernandez-Diaz S, Huybrechts KF, Fischer MA, Creanga AA, Callaghan WM, Gagne JJ (2013). Development of a comorbidity index for use in obstetric patients. Obstet Gynecol.

[18] Chuang CH, Hsieh WS, Guo YL, Tsai YJ, Chang PJ, Lin SJ, Chen PC (2007). Chinese herbal medicines used in pregnancy: a population-based survey in Taiwan. Pharmacoepidemio Drug Saf.

[19] Nordeng H, Bayne K, Havnen GC, Paulsen BS (2011). Use of herbal drugs during pregnancy among 600 Norwegian women in relation to concurrent use of conventional drugs and pregnancy outcome. Complement Ther Clin Pract.

[20] Zhu X, Qi X, Hao J, Huang Z, Zhang Z, Xing X, Cheng D, Xiao L, Xu Y, Zhu P (2010). Pattern of drug use during the first trimester among Chinese women: data from a population-based cohort study. Eur J Clin Pharmacol.

[21] Hollyer T, Boon H, Georgousis A, Smith M, Einarson A (2002). The use of CAM by women suffering from nausea and vomiting during pregnancy. BMC Complement Altern Med.

[22] Thomson M, Corbin R, Leung L (2014). Effects of ginger for nausea and vomiting in early pregnancy: a meta-analysis. J Am Board Fam Med.

[23] Wang C, Wang H, Liu X, Xu D, Tang Y, Luo P (2014). Traditional Chinese medicine for the treatment of influenza: a systematic review and meta-analysis of randomized controlled trials. J Tradit Chin Med.

[24] Chu MHK, Wu IXY, Ho RST, Wong CHL, Zhang AL, Zhang Y, Wu JCY, Chung VCH (2018). Chinese herbal medicine for functional dyspepsia: systematic review of systematic reviews. Ther Adv Gastroenterol.

[25] Soma-Pillay P, Nelson-Piercy C, Tolppanen H, Mebazaa A (2016). Physiological changes in pregnancy. Cardiovas J Afr.

[26] Washio M (2003). Kami-shoyo-san is usually used for women. Psychiatry Clin Neurosci.

[27] Park DM, Kim SH, Park YC, Kang WC, Lee SR, Jung IC (2014). The comparative clinical study of efficacy of Gamisoyo-San (Jiaweixiaoyaosan) on generalized anxiety disorder according to differently manufactured preparations: multicenter, randomized, double blind, placebo controlled trial. J Ethnopharmacol.

[28] Park SW, Kim YK, Lee JG, Kim SH, Kim JM, Yoon JS, Park YK, Lee YK, Kim YH (2007). Antidepressant-like effects of the traditional Chinese medicine kami-shoyo-san in rats. Psychiatry Clin Neurosci.

[29] Mizowaki M, Toriizuka K, Hanawa T (2001). Anxiolytic effect of Kami-Shoyo-San (TJ-24) in mice: possible mediation of neurosteroid synthesis. Life Sci.

[30] Cao GP, Gui D, Fu LD, Guo ZK, Fu WJ (2016). Anxiolytic and neuroprotective effects of the traditional Chinese medicinal formulation Dan-zhi-xiao-yao-san in a rat model of chronic stress. Mol Med Rep.

[31] Xiao Y, Liu YY, Yu KQ, Ouyang MZ, Luo R, Zhao XS (2012). Chinese herbal medicine liu jun zi tang and xiang sha liu jun zi tang for functional dyspepsia: meta-analysis of randomized controlled trials. Evid Based Complement Alternat Med.

[32] Zhong XH, Zhou ZX, Li TS, Wang EQ, Shi WY, Chu SM (2002). Anti-abortive effect of Radix scutellariae and Rhizoma atractylodis in mice. Am J Chin Med.

[33] Hussain T, Tan B, Liu G, Oladele OA, Rahu N, Tossou MC, Yin Y (2016). Health-promoting properties of Eucommia ulmoides: a review. Evid Based Complement Alternat Med.

[34] Crespin S, Bourrel R, Hurault-Delarue C, Lapeyre-Mestre M, Montastruc JL, Damase-Michel C (2011). Drug prescribing before and during pregnancy in south West France: a retrolective study. Drug Saf.

[35] Kao LT, Chen YH, Lin HC, Chung SD (2014). Prescriptions for category D and X drugs during pregnancy in Taiwan: a population-based study. Pharmacoepidemiol Drug Saf.

[36] Pritchard AM, Hendrix PW, Paidas MJ (2016). Hereditary thrombophilia and recurrent pregnancy loss. Clin Obstet Gynecol.

[37] Santos TDS, Ieque AL, de Carvalho HC, Sell AM, Lonardoni MVC, Demarchi IG, de Lima Neto QA, Teixeira JJV (2017). Antiphospholipid syndrome and recurrent miscarriage: a systematic review and meta-analysis. J Reprod Immunol.

[38] Ziakas PD, Pavlou M, Voulgarelis M (2010). Heparin treatment in antiphospholipid syndrome with recurrent pregnancy loss: a systematic review and meta-analysis. Obstet Gynecol.

[39] Taiwan Herbal Pharmacopeia 3rd Edition Committee. Taiwan Herbal Pharmacopeia 3rd Edition English version. Taiwan, R.O.C: Ministry Health and Welfare; 2019.

[40] Zhu YZ, Wu W, Zhu Q, Liu X (2018). Discovery of Leonuri and therapeutical applications: from bench to bedside. Pharmacol Ther.

[41] Lee H, Choi TY, Myung CS, Lee JA, Lee MS (2016). Herbal medicine (Shaofu Zhuyu decoction) for treating primary dysmenorrhea: a systematic review of randomized clinical trials. Maturitas.

[42] Zhu G, Jiang C, Yan X, Zhao S, Xu D, Cao Y (2018). Shaofu Zhuyu decoction regresses Endometriotic lesions in a rat model. Evid Based Complement Alternat Med.

