# Supplementary material for: Prescription patterns and factors influencing the use of Chinese herbal medicine among pregnant women in Taiwan: a population-based retrospective study
Source: BMC Complement Med Ther. 2020 Jul 30;20:240. doi: 10.1186/s12906-020-03032-0 (PMC7391530; doi:10.1186/s12906-020-03032-0)
Supplement: Supplementary file 1 — Additional file 1: Table S1. Top 10 diagnosis and prescription in CHM users during pregnancy during follow up. Table S2. The ICD-9-CM codes for maternal comorbidities. [file 12906_2020_3032_MOESM1_ESM.docx]

**Supplementary Table 1** Top 10 diagnosis and prescription in CHM users during pregnancy during follow up

| Diagnosis | Frequency (%)^1^ | Single herbal (Latin name) | Frequency (%)^2^ | Herbal formulae  (Pin-yin name) | Frequency (%)^3^ |
| --- | --- | --- | --- | --- | --- |
| Abnormal bleeding from female genital tract | 33766 (12.0) | *Scutellariae Radix* (Huang Qin) | 7125 (4.4) | Dang-Gui-Shao-Yao-San | 4948 (4.1) |
| Acute nasopharyngitis | 31437 (11.2) | *Eucommiae cortex* (Du Zhong) | 4010 (2.5) | Jia-Wei-Xiao-Yao-San | 4203 (3.5) |
| Cough | 14574 (5.2) | *Atractylodes Rhizome* (Bai Zhu) | 3929 (2.4) | Xiang-Sha-Liu-Jun-Zi-Tang | 3113 (2.6) |
| Disorders of function of stomach | 12737 (4.5) | *Cyperi Rhizoma* (Xiang Fu) | 3384 (2.1) | Bao-Chan-Wu-You-Fang | 3076 (2.6) |
| Hypertension complicating pregnancy, childbirth, and the puerperium | 10647 (3.8) | *Cuscutae Semen* (Tu Si Zi) | 3204 (2.0) | Yin-Qiao-San | 2656 (2.2) |
| Excessive vomiting in pregnancy | 10568 (3.8) | *Dipsaci Radix* (Xu Duan) | 3161 (2.0) | Xin-Yi-Qing-Fei-Tang | 2391 (2.0) |
| Headache | 10229 (3.7) | *Platycodi Radix* (Jie Geng) | 2846 (1.8) | Ban-Xia-Xie-Xin-Tang | 2162 (1.8) |
| Constipation | 9914 (3.5) | *Corydalis Rhizoma* (Yan Hu Su) | 2585 (1.6) | Ping-Wei-San | 2106 (1.8) |
| Sleep disturbances | 9793 (3.5) | *Fritillariae Thunbergii Bulbus* (Bei Mu) | 2567 (1.6) | Chuan-Qiong-Cha-Tiao-San | 2104 (1.8) |
| Allergic rhinitis | 7606 (2.7) | *Glycyrrhizae Radix et Rhizoma* (Gan Cao) | 2491 (1.6) | Xiao-Chai-Hu-Tang | 1952 (1.6) |

^1^the total number of diagnosis is 280,397; ^2^ the total number of prescriptions for single herbal is 160,973; ^3^ the total number of prescriptions for herbal formulae is 119,591

**Supplementary Table 2** The ICD-9-CM codes for maternal comorbidities.

| Comorbidity | ICD-9-CM code |
| --- | --- |
| Gestational hypertension | 642.3 |
| Congenital heart disease | 745-747.4, 648.5 |
| Chronic ischemic heart disease | 412- 414 |
| Cardiac valvular disease | 394-397, 424 |
| Preexisting hypertension | 401-405, 642.0-642.2 |
| Sickle cell disease | 282.4, 282.6 |
| Placenta previa | 641.0, 641.1 |
| Chronic renal disease | 581-583, 585, 587, 588, 646.2 |
| Asthma | 493 |
| Preexisting diabetes mellitus | 250, 648.0 |
